# Retracted: Design and Workspace Analysis of a Parallel Ankle Rehabilitation Robot (PARR)

**DOI:** 10.1155/2021/7345780

**Published:** 2021-01-28

**Authors:** Journal of Healthcare Engineering

The article titled “Design and Workspace Analysis of a Parallel Ankle Rehabilitation Robot (PARR)” [1] has been retracted at the request of the authors after they found errors in the kinematic analysis of the PARR.

The authors say that the 2-UPS/RRR configuration was used in the PARR, with joints S and U taken as independent joints and the corresponding centers simplified as single points in Figure 6. The spherical joints S1 and S2 were composed of three rotational joints, and the three centers of the rotational joints did not coincide at one point. Similarly, the universal joints U1 and U2 were composed of two rotational joints distributed vertically, and the distance between the centers of the rotational joints should not have been neglected. These simplifications lead to incorrect results of the theoretical workspace. If the simplification is corrected, this provides more accurate results though the error is less than 10%.

In addition, the simplifications were not helpful for precise motion control. During the measurement of the effective workspace (EWS) of PARR, although the thigh and calves were tied and fixed with wooden clamps using elastic straps, the thigh and calves still moved slightly and influenced the results. It will be necessary to attach an inertial attitude sensor on the knee to eliminate the influence of the movements and give the confidence interval of the EWS. Hence, the workspace analysis of PARR was insufficient, and a more precise model should be developed.

For the first issue, the corresponding details were stated in the second and fourth paragraphs of Section 3, “Kinematic Analysis of PARR.” For the second issue, the corresponding details were stated in the second paragraph of Section 4.1, “PMS of Ankle Joint.” The two issues affected the results depicted in Figures 8, 9, 11, and 13.

The editor noted that the assumptions made by the authors in the published article were plausible, though the validity of the simplified model was not thoroughly verified.
